# Epidemiology of rotavirus infection among young children with acute diarrhoea in Burkina Faso

**DOI:** 10.1186/1471-2431-10-94

**Published:** 2010-12-20

**Authors:** Isidore JO Bonkoungou, Idrissa Sanou, Fabienne Bon, Benoit Benon, Sheick O Coulibaly, Kaisa Haukka, Alfred S Traoré, Nicolas Barro

**Affiliations:** 1 Laboratoire de Biochimie et Biologie Moléculaire, CRSBAN/UFR-SVT, Université de Ouagadougou, 03 BP 7021, Ouagadougou, Burkina Faso; 2 Laboratoire National de Santé Publique, 09 BP 24, Ouagadougou, Burkina Faso; 3 Laboratoire de Bactériologie et Virologie du CHU-Yalgado Ouédraogo, 03 BP 7022 Ouagadougou, Burkina Faso; 4 UFR Sciences de la Santé, Université de Ouagadougou, 03 BP 7021, Ouagadougou, Burkina Faso; 5 Laboratoire Interactions Muqueuses-Agents Transmissibles-UFR Médecine, 21079 Dijon, France; 6 Service de pédiatrie, Centre Médical du Secteur 30 de Ouagadougou, Ouagadougou, Burkina Faso; 7 Department of Infectious Disease Surveillance and Control, Bacteriology Unit, National Institute for Health and Welfare, P.O. Box 30, 00271 Helsinki, Finland

## Abstract

**Background:**

In anticipation of vaccine introduction, we assessed epidemiology of rotavirus disease among children visiting medical centre due to acute diarrhoea in Ouagadougou, Burkina Faso.

**Methods:**

Between November 2008 and February 2010, stool specimens from 447 children less than 5 years of age suffering from diarrhoea were tested for the presence of rotavirus by antigen detection using an immunochromatographic test. Sociodemographic, environmental and clinical factors were assessed during the study.

**Results:**

Rotavirus antigen was detected in 151 (33.8%) of the patients. Most of the cases (94.2%) were in children < 24 months of age. Fever and vomiting were the symptoms most commonly reported in association with rotavirus diarrhoea and the patients were often hospitalized. Rotavirus-associated diarrhoea occurred mostly during the season from December to April (dry season). Rotavirus infection was significantly less frequent in breast-fed than among bottle-fed babies.

**Conclusions:**

The results of this study underscore the need to control rotavirus infections among young children in Burkina Faso and may argue a decision on the introduction of rotavirus vaccine in Burkina Faso.

## Background

Rotavirus is a major cause of acute gastroenteritis in infants and young children worldwide [1]. It has been estimated that about 39% of childhood diarrhoea hospitalizations are caused by rotaviruses and nearly half a million children die from rotavirus infections each year [2]. Furthermore, rotavirus mortality is concentrated in the developing countries on the Asian subcontinent, Africa, and Latin America where access to health care facilities is limited [3]. This may result in a significant disease burden and economic effect of direct medical costs, loss of work, quality of life and mortality. In other diarrhoeal diseases, improvement of hygiene and sanitation may reduce the incidence, but these measures are unlikely sufficient for rotavirus control. Vaccination is the only control measure likely to have a significant impact on the incidence of severely dehydrating rotavirus disease [4]. Two new live-attenuated rotavirus vaccines (Rotarix^® ^and RotaTeq^®^) have demonstrated very good safety and efficacy profiles in large clinical trials in the Western industrialized countries and in Latin America [5-9]. Trials on these vaccines are now in progress in sub-Saharan Africa to assess their effectiveness and efficacy.

In Burkina Faso, very few data on illness caused by rotavirus have been published and these studies indicated that 14% of acute diarrhoea in children under the age of 5 years is due to rotavirus infection but epidemiological data are still incomplete [10].

The objectives of this study were to describe, for the first time, epidemiology of rotavirus disease among children visiting a local health centre because of acute gastroenteritis in Ouagadougou in Burkina Faso to provide background knowledge on the disease before vaccine introduction and to inform the policy makers on the need for the introduction of new rotavirus vaccines.

## Methods

### Study population and specimens

The study was conducted at Centre Médical avec Antenne Chirugicale (CMA) du Secteur 30 in the capital city of Ouagadougou, Burkina Faso. CMA du secteur 30, located in the Bogodogo district is one of the four secondary health care centers in Ouagadougou and its paediatric ward has a capacity of 30 beds and admits over 2260 children each year. Ouagadougou has a population of nearly two millions, whereas the Bogodogo district has a population of about 548000 with 81000 (15%) children < 5 years of age http://www.sante.gov.bf. The study protocol was approved by the Ethics Committee of Burkina Faso. Parents of all the paediatric patients were informed on the study details and their oral consent was obtained before stool specimen and epidemiological data collection during the course of treatment. Written consent was obtained from parents of the control group. All children under the age of 5 year visiting the paediatric service for treatment of gastroenteritis from November 2008 to February 2010 were included in the study. Diarrhoea was defined as the passage of three or more loose or watery stools in the preceding 24 h. During this study, 471 patients were included as soon as they were seen by a physician and fresh stool samples were collected and transferred to the Microbiology Laboratory at the National Public Health Laboratory, Ouagadougou, for rotavirus and adenovirus detection. Control stool samples were collected from 60 randomly selected children coming to the same health centre for routine immunization and not presenting gastroenteritis symptoms. The age of the control was paired with the patient's age. Information regarding the age, sex, type of nutrition (breast-fed and/or bottle-fed), hospitalization and clinical symptoms such as fever, vomiting and dehydration, and the characteristics of stool were recorded for each child. Also hygiene factors such as source of drinking water of the child were collected during the study.

### Detection of rotavirus in stool samples

All stool samples were analyzed for group A rotavirus using one step rotavirus and adenovirus serotypes 40/41 test for determination of rotavirus and adenovirus in human feces (SD Bioline Rota/Adeno^®^; Standard diagnostics, Inc., Korea) following the manufacturer's instructions.

### Statistical analyses

χ^2 ^test was used to analyze the data and the p value less than 0.05 was considered statistically significant.

## Results

### Rotavirus prevalence

Out of the 471 children with acute diarrhea initially included in the study, 24 were subsequently excluded because for 19 of them no sample was collected and for 5 of them no epidemiological data were available. Of the 447 stool specimens analysed, 151 (33.8%) were found to contain rotavirus. Only one (1.7%) out of the 60 stool specimens collected from healthy children was positive by immunochromatographic test (ICG) (p < 0.0001). Adenovirus was detected in 17 of 447 (3.8%) stool samples and mixed infections with both rotavirus and adenovirus were observed in 11 (2.5%) stool samples.

### Age and sex distribution of patients with rotavirus infection

**Figure 1:**
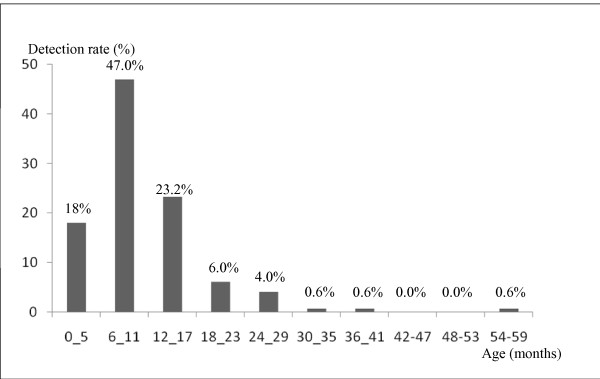
**Proportion of rotavirus infections by age groups among the 447 children suffering of gastroenteritis, between November 2008 to February 2010**.

The age distribution of children with rotavirus is shown in Figure 1. Most cases of rotavirus infection (94.2%) occurred among children less than 2 years of age. The highest incidence was observed in children between 6 and 11 months of age. The median age for rotavirus infection was 8 months. There were more males (52.8%) than females, but the sex ratio among the rotavirus diarrhea patients was not significant (p = 0.1).

### Seasonal distribution of rotavirus infections

**Figure 2:**
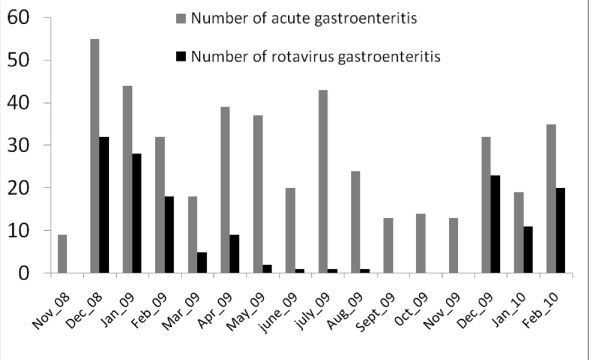
**Seasonality of the acute gastroenteritis and the rotavirus infections among the 447 children suffering of gastroenteritis, between November 2008 to February 2010**.

During the whole period of surveillance, the incidence of rotavirus infections varied significantly according to seasons (warm and cold) (p = 0.0001). Rotavirus infections occurred mostly during the season from December to April, corresponding to the dry season and relatively cold period (Figure 2).

### Hospitalization and clinical presentation of rotavirus infection

Of the 217 outpatient children, 48 (22.1%) were infected and of the 230 inpatient children, 103 (44.8%) were infected with rotavirus. The rotavirus infection prevalence was significantly higher among hospitalized children (p = 0.0001) illustrating a significant relationship between rate of hospitalization - severity of illness and diarrhoea associated with rotavirus infection. In addition, fever was the symptom most commonly reported in association with rotavirus diarrhoea (82.1%), followed by vomiting (72.8%) and dehydration (48.3%) (Table 1).

**Table 1:** Clinical and epidemiological features of children with and without rotavirus detected in the diarrheal stool sample.

Epidemiological and clinical characteristics		Rotavirus Diarrhea	Non Rotavirus Diarrhea	p
		(N = 151)	(N = 296)	
Sex				
	Male	71 (47.0%)	165 (55.7%)	p = 0.1
	Female	80 (53.0%)	131 (44.8%)	
Mean age (months)		10.9	16.6	
Patient status				
	Inpatient	103 (44.8%)	127 (55.2%)	p = 0.0001
	Outpatient	48 (22.1%)	169 (77.9%)	
Symptom				
	Fever	124 (82.1%)	161 (54.4%)	p = 0.0001
	Vomiting	110 (72.8%)	80 (27.0%)	p = 0.0001
	Dehydration	73 (48.3%)	54 (18.2%)	p = 0.0001
Season*				
	Warm	5 (2.9%)	168 (97.1%)	p = 0,0001
	Cold	146 (53.3%)	128 (46.7%)	
Breast feeding**				
	Yes	59 (59.0%)	105 (75.5%)	p = 0.010
	No	41 (41.0%)	34 (24.5%)	
Drinking water source				
	Municipal supply	108 (71.5%)	142 (48.0%)	p = 0.0001
	Bottled water	43 (28.5%)	154 (52.0%)	

*Percentage of rotavirus diarrhea versus non-rotavirus diarrhea for both seasons.

**Percentage of rotavirus diarrhea versus non-rotavirus diarrhea according to method of feeding among infants ≤ 9 months of age.

### Nutrition and drinking water

Among the infants ≤ 9 months of age, who had rotavirus diarrhoea and for whom the method of feeding was recorded, 55 out of 154 were breast-fed (35.7%) and 32 out of 61 were bottle-fed (52.5%) (p = 0.01). Analysis of hygiene factors such as the source of drinking water showed that children drinking municipal water were more affected by rotavirus diarrhoea than children drinking mineral water sold in bottles (p = 0.0001) (Table 1).

## Discussion

This was the first study in Burkina Faso to investigate the prevalence, clinical characteristic and risk factors of rotavirus gastroenteritis among children. Regardless our results, rotavirus diarrhoea appears to be a major public health problem for children in Burkina Faso, as in the other developing countries. Our results show that a significant proportion of acute diarrhoea is due to rotavirus (33.8%) and rotavirus may be responsible for almost one-half (44.8%) of all hospitalizations for diarrhea in children < 5 years of age in Burkina Faso. The detected prevalence appears to be similar to those reported from other West African countries, which ranged from 33% to 39% [11-13]. In addition, a cumulative experience from 15 African countries suggested that rotavirus is the most important cause of severe diarrhoea in African children [14].

As observed in the other parts of the world, the burden of rotavirus disease is predominantly borne by children less than 2 years of age [15] with a high incidence among children 6-11 months of age. This can be explained by the protective effect of maternal antibodies in < 6 months old, and the development of natural immunity after repeated infections in children over 2 years of age [16,17].

Our results showed that rotavirus occurred mostly during the season from December to April, corresponding to the dry season and relatively cold period, as has been reported from Northern Ghana near Burkina Faso [13] and Guinea-Bissau [11]. Some studies conducted in other African countries indicated that rotavirus infections are present throughout the year, but with much higher prevalence in a certain period of a year [14].

Comparison of the clinical characteristics and severity of the acute gastroenteritis among the rotavirus-positive and rotavirus-negative patients indicated that vomiting, fever and dehydration were more frequently observed among diarrheal children with rotavirus than among those without rotavirus infection, as reported in the other countries [11,18].

Our confirmation of a previous observation made in the other parts of the world that during the first year of life breastfeeding is associated with a lower incidence of rotavirus diarrhoeal episodes adds to the multitude of benefits that have been associated with breastfeeding [19,20]. In addition, it has been shown that even if the breast-fed infants get infected with rotavirus, a milder disease occurs and hospitalization rate is significantly lower [21].

Another important issue, which was shown in this study, is a significant association between rotavirus diarrhea and municipal drinking water. This may be due to the possible contamination of municipal water for human consumption or in inter-human contamination which drinking water may be a potential risk of rotavirus transmission. Rotavirus has been described as a causative agent in several waterborne outbreaks in the industrialized countries [22-25], indicating good survival of rotavirus in water. In Burkina Faso, the evaluation of drinking water quality does not require testing for rotavirus but our results show the importance of including routine virological analysis of drinking water during rotavirus season.

## Conclusions

In conclusion, this study provides information on the epidemiology and the extent of rotavirus infections in Burkina Faso. Our results indicate that gastroenteritis caused by rotavirus in the country is an important health problem, particularly among children less than 2 years of age and during the cold season. These data will be useful for making an informed decision about the introduction of rotavirus vaccine in Burkina Faso and will provide a baseline against which the impact of the vaccine introduction can be measured in the future.

## Competing interests

The authors declare that they have no competing interests.

## Authors' contributions

IJOB, IS and NB conceived the study; BB and IJOB were in charge of recruitment, examination, treatment and follow-up of patients, controls and undertook laboratory analysis; IJOB, IS and NB analyzed the data and prepared the manuscript; NB, IS, FB, SOC, KH and AST secured the study execution and provided ideas and comments during manuscript preparation. All authors have read and approved the final manuscript.

## Pre-publication history

The pre-publication history for this paper can be accessed here:

http://www.biomedcentral.com/1471-2431/10/94/prepub
